# Quantitative Genetics Identifies Cryptic Genetic Variation Involved in the Paternal Regulation of Seed Development

**DOI:** 10.1371/journal.pgen.1005806

**Published:** 2016-01-26

**Authors:** Nuno D. Pires, Marian Bemer, Lena M. Müller, Célia Baroux, Charles Spillane, Ueli Grossniklaus

**Affiliations:** Department of Plant and Microbial Biology and Zurich-Basel Plant Science Center, University of Zurich, Zürich, Switzerland; Georgia Institute of Technology, UNITED STATES

## Abstract

Embryonic development requires a correct balancing of maternal and paternal genetic information. This balance is mediated by genomic imprinting, an epigenetic mechanism that leads to parent-of-origin-dependent gene expression. The parental conflict (or kinship) theory proposes that imprinting can evolve due to a conflict between maternal and paternal alleles over resource allocation during seed development. One assumption of this theory is that paternal alleles can regulate seed growth; however, paternal effects on seed size are often very low or non-existent. We demonstrate that there is a pool of cryptic genetic variation in the paternal control of *Arabidopsis thaliana* seed development. Such cryptic variation can be exposed in seeds that maternally inherit a *medea* mutation, suggesting that MEA acts as a maternal buffer of paternal effects. Genetic mapping using recombinant inbred lines, and a novel method for the mapping of parent-of-origin effects using whole-genome sequencing of segregant bulks, indicate that there are at least six loci with small, paternal effects on seed development. Together, our analyses reveal the existence of a pool of hidden genetic variation on the paternal control of seed development that is likely shaped by parental conflict.

## Author Summary

In plants and mammals, embryo development occurs under the protection and nourishment of maternal tissues. In polygamous species, this can lead to competition between siblings for privileged access to maternal nutrients. According to the parental conflict theory—a variation of the kinship theory—the asymmetric genetic relatedness between offspring from multiple fathers may lead to the evolution of parent-of-origin-dependent developmental regulation; paternally inherited alleles would benefit from maximizing embryo growth (at the expense of siblings), whereas maternally inherited alleles would benefit from restraining growth (to equalize sibling resource allocation). The kinship theory assumes that the paternal genome can actually influence embryo development; however, plant seed development is under strong maternal control. Here, we show that there is a hidden pool of variation in paternal effect loci that can be released upon loss of *MEDEA*, a major maternal regulator of seed development. Our results demonstrate that the maternal genome actively buffers the effects of paternal genomes on seed development, thereby providing strong functional support to the parental conflict theory.

## Introduction

Post-fertilisation development is a complex process that involves dynamic interactions between maternally and paternally derived genomes. A correct balancing of parental genomes is essential for embryonic development, and disruptions of this balance (e.g. by crossing individuals with different ploidies) often lead to embryo inviability [1–6]. Genomic imprinting, an epigenetic mechanism that leads to differential expression of alleles in a parent-of-origin-dependent manner, is responsible for many parental asymmetries during embryo and seed development in mammals and flowering plants [7,8].

Transcriptome profiling of developing seeds has revealed the existence of hundreds of candidate imprinted genes in the embryo and/or endosperm, a biparental nourishing tissue that derives from a second fertilisation event (reviewed in [9–11]). However, the functional role of genomic imprinting is still a matter of considerable theoretical debate [12]. The parental conflict (or kinship) theory of genomic imprinting proposes that imprinting can evolve as the manifestation of a conflict of interests between maternal and paternal alleles over resource allocation during embryogenesis or seed development [13–15]. This conflict arises due to the asymmetric genetic relatedness between maternal and paternal alleles in polyandrous (multiple paternity) species, where maternal alleles are more likely to be shared between siblings than paternal alleles.

The parental conflict theory is supported not only by mutant phenotypes in mice [16–18] but also by the discovery of *MEDEA* (*MEA*), a major regulator of imprinting and the maternal control of seed development in *Arabidopsis thaliana* (L.) Heynh (referred to as *Arabidopsis* hereafter). Only the maternal *MEA* allele is expressed (before fertilization in the embryo sac that contains the female gametes and later in the embryo and endosperm derived from these gametes) [19,20], and seeds that maternally inherit a loss-of-function *mea* allele undergo excessive cell proliferation and eventually abort [21]. *MEA* encodes a SET-domain histone methyltransferase that catalyses the trimethylation of H3K27—a repressive epigenetic mark associated with gene silencing—as part of a seed-specific version of the *Polycomb* Repressive Complex 2 (FIS-PRC2) [22]. This suggests that an important function of MEA is to maternally restrain seed growth by negatively regulating the expression of genes that would otherwise promote embryo and endosperm growth.

While the parental conflict theory predicted the existence of maternal regulators of seed development such as *MEDEA (MEA)*, the paternal genotype has no or very small effects on seed growth [23–30]. Here we show that there is a large hidden pool of natural variation in the paternal control of seed development that can be exposed using a maternal mutant *mea* background. Using a combination of classic quantitative trait analysis and a novel method for whole-genome sequencing of bulk segregants (Bulk-Seq), we determined that at least six loci contribute to the paternal rescue of *mea* seeds. Together, our results indicate that there is a large pool of natural variation in loci exerting paternal effects on seed development in *Arabidopsis*. These paternal effects are buffered by maternal *MEA* activity, suggesting that they were likely shaped by parental conflict.

## Results

### *mea* seeds can be paternally rescued

**Fig 1:**
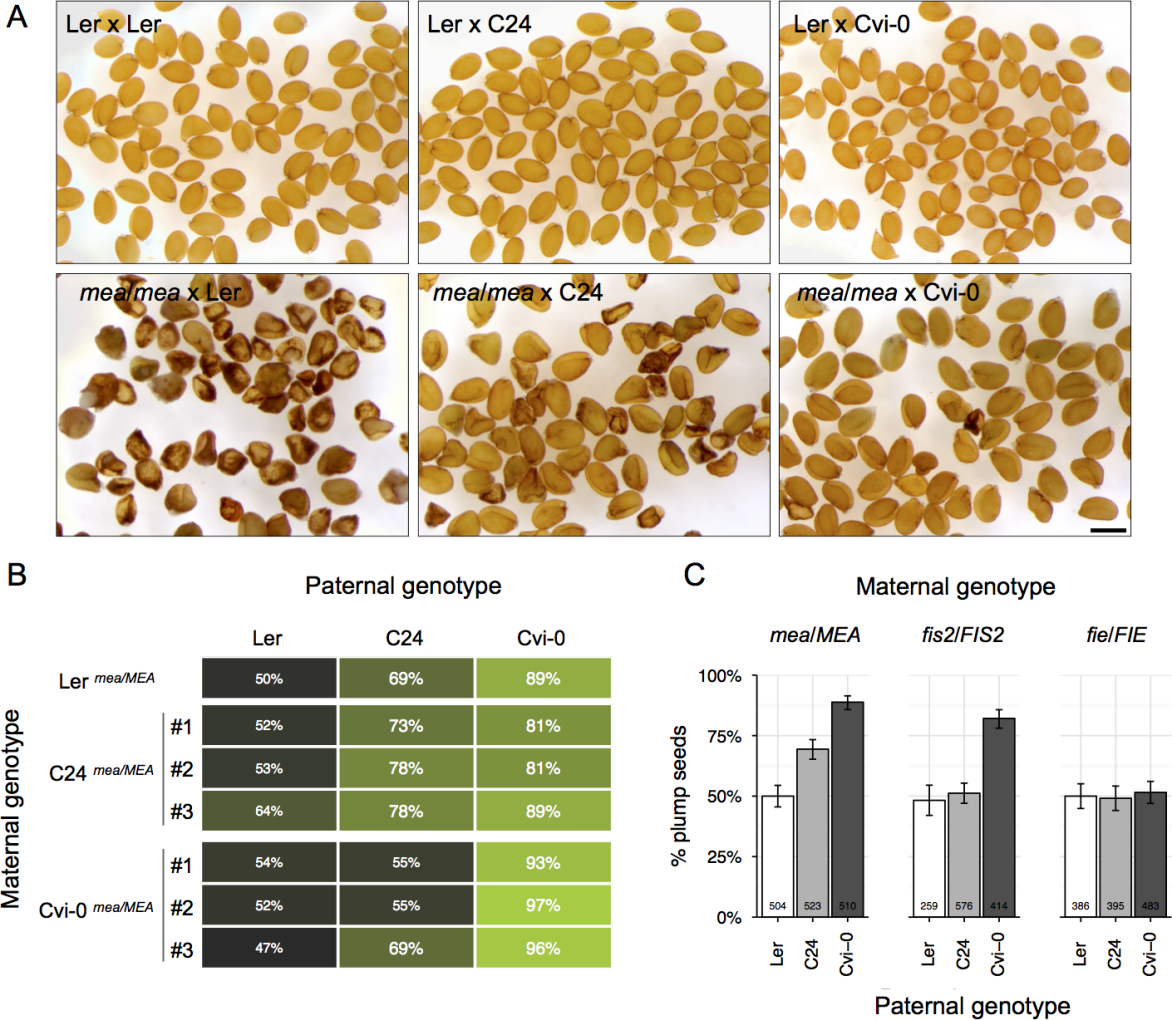
*mea* seed abortion can be paternally suppressed. (A) Mature F1 seeds derived from crosses between Ler or homozygous *mea-1*/*mea-1* and pollen from Ler, C24, and Cvi-0. The scale bar represents 500μm. (B) Percentage of viable plump seeds derived from crosses between heterozygous *mea-2*/*MEA* introgressed into C24 and Cvi-0 backgrounds and pollen from Ler, Cvi-0, and C24. Three independent backcross lineages were generated for each accession, and one maternal individual per lineage was assayed. (C) Percentage of viable plump seeds derived from crosses between heterozygous *mea-2*/*MEA*, *fis2*/*FIS2* and *fie*/*FIE* and pollen from Ler, Cvi-0, and C24. The numbers in the bars indicate the number of seeds sampled; the error bars denote 95% binomial confidence intervals.

When *mea* ovules are pollinated with wild-type pollen from the Landsberg *erecta* accession (hereafter referred to as Ler), seeds undergo excessive cell proliferation and abort before completing embryogenesis [21]. However, *mea* ovules pollinated with pollen from other *Arabidopsis* accessions (such as Cvi-0 or C24) can give rise to viable plump *mea* seeds (Fig 1A and S1 Fig). To dissect the relative paternal and maternal contributions to *mea* seed rescue, we introgressed *mea-2* (originally in the Ler background) into Cvi-0 and C24. After six generations of backcrossing, we crossed three independent Cvi-0^*mea/MEA*^ and C24^*mea/MEA*^ lines with pollen from Ler, C24, and Cvi-0: all the pollinations made with Ler pollen resulted in high rates of seed abortion, whereas the pollinations made with Cvi-0 or C24 resulted in mostly viable plump seeds, independently of the genotype of the maternal plant used (Fig 1B). The magnitude of the Cvi-0 and C24 paternal rescue was modulated by the maternal genotype (e.g. in a Cvi-0^*mea/MEA*^ maternal background the paternal effect of Cvi-0 was stronger and the effect of C24 was very weak). This suggests that the rescue of *mea* seeds is primarily a paternal-specific effect that can be partially modulated by the maternal genotype, indicating the existence of strong reciprocal interactions between the two parental genomes.

Since *MEA* is an imprinted gene (only its maternal allele is expressed) a potential explanation for *mea* seed rescue could be an activation of the paternal wild-type *MEA* allele. However, this hypothesis cannot easily be tested using allele-specific expression assays, because maternal *mea* mutations already induce low levels of paternal *MEA* expression [20,31–33]. To determine genetically if the paternal *MEA* allele is required for *mea* seed rescue, we examined the F2 progeny of crosses between *mea-2* and different *Arabidopsis* accessions. If a paternal *MEA* allele was required for the rescue, we would not expect to recover viable homozygous *mea/mea* seeds. However, we recovered 9–20% of viable *mea/mea* plants in the F2 progeny of crosses with the accessions C24, Hs-0, and Lomm1-1 (S1 Table). This result clearly indicates that a paternal *MEA* allele is not required for *mea* seed rescue in these crosses. In the crosses with Cvi-0 we only recovered 3% of homozygous *mea/mea* seeds; when these different F2 homozygous Cvi-0^*mea/mea*^ individuals were self-fertilized, however, we observed a range of 1–60% plump F3 seeds in their progeny (S2 Fig). This finding suggests that in *mea* crosses with Cvi-0, the paternal *MEA* allele (or a closely linked locus) can enhance but is not required for the rescue of *mea* seeds.

*MEA* encodes a subunit of the FIS-PRC2 complex, which also contains the zinc finger protein *FERTILIZATION-INDEPENDENT SEED2* (*FIS2*) and the WD40 domain protein *FERTILIZATION-INDEPENDENT ENDOSPERM* (*FIE*) [22]. To test whether Cvi-0 and C24 can also rescue seed abortion caused by mutations in these genes, we crossed heterozygous *fis2/FIS2* and *fie/FIE* plants with pollen from Ler, Cvi-0, and C24 (Fig 1C). While Cvi-0 could rescue *fis2* seeds, there was no significant seed rescue using C24 pollen. *fie* seeds could not be rescued by pollen of either Cvi-0 or C24. These results indicate that the *mea* seed paternal rescue does not simply occur at the FIS-PRC2 level; rather it supports the hypotheses that the different FIS-PRC2 subunits play distinct roles [34] and that MEA participates in multiple protein complexes during seed development [32].

### There is extensive variation in the penetrance of *mea* in different *Arabidopsis* accessions

**Fig 2:**
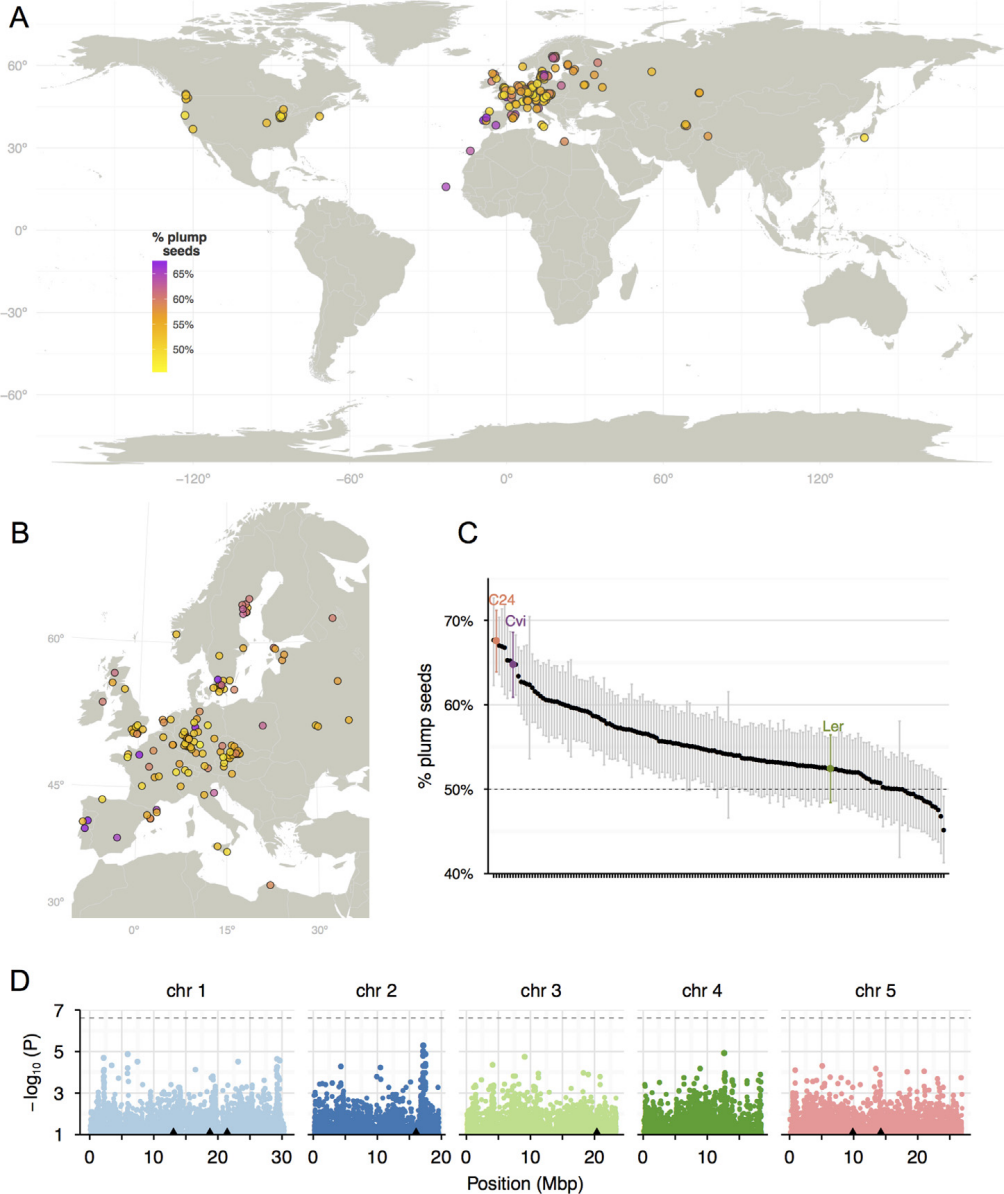
Cryptic genetic variation for parent-of-origin effects during *Arabidopsis* seed development. (A-C) Percentage of viable plump seeds in the F2 progeny of *mea-2* with 164 *Arabidopsis* accessions. See also S2 Table. Error bars indicate 95% binomial confidence intervals. (D) Manhattan plots indicating the significance of associations between variation at SNPs across the genome and *mea* rescue. The black triangles indicate the position of the peaks from the Bulk-Seq analysis. The dashed line indicates the 5% significance threshold with Bonferroni correction.

To determine the extent of species-wide variation on *MEA*-dependent parental interactions, we pollinated 164 *Arabidopsis* accessions with *mea-2* to generate F1s that were allowed to self-fertilize to examine seed viability rates in the F2 generation. Each of the F2 populations segregates (1) *MEA* and *mea*, and (2) chromosomes from Ler and the respective parental accessions: therefore, we expected to obtain 50% viable seeds from accessions that do not modify the penetrance of *mea* (such as Ler), and up to 75% viable seeds from accessions with a strong paternal rescue effect (assuming no epistatic effects). Accordingly, we observed 52% plump seeds in the control Ler crosses, while in the Cvi-0 and C24 crosses there were 65% and 68% plump seeds, respectively (Fig 2A–2C). Roughly half of the accessions tested showed between 55% and 70% plump seeds, suggesting that alleles that modify the penetrance of *mea* seed abortion are widespread among natural *Arabidopsis* accessions.

Half of the strongest *mea* rescuers originate from latitudes more southern than 45° (Fig 2A), but we found no significant linear correlation between the geographical origin of the accessions and their effect on the penetrance of *mea*. We compared the *mea* rescue effect of these accessions with over 100 phenotypes reported for a large set of *A*. *thaliana* accessions [35]. The only statistically significant traits correlated with the rescue of *mea* were *in planta* magnesium and calcium concentrations (Pearson correlation, 5% false discovery rate) (S3 Fig). We also found evidence for an association between flowering time and *mea* rescue, as half of the strongest *mea* rescuers included very early flowering accessions under field or short day conditions (Cvi-0, C24, Se-0, Ts-1 and Co) (S3 Fig). We did not find a correlation between *mea* rescue and the size of self-fertilized or outcrossed *Arabidopsis* seeds [30,36].

We performed a genome-wide association study (GWAS) to identify regions in the genome whose species-level variation is linked to *mea* rescue. However, we were unable to detect clear statistically significant associations (Fig 2D), likely due to the weak power of GWAS to detect polygenic traits with low effect sizes [37]. Nevertheless, some of the most highly associated SNPs were in the vicinity of the regions identified with the Bulk-Seq analysis (see below).

### QTL analyses identify six loci that contribute to the rescue of *mea* seeds

**Fig 3:**
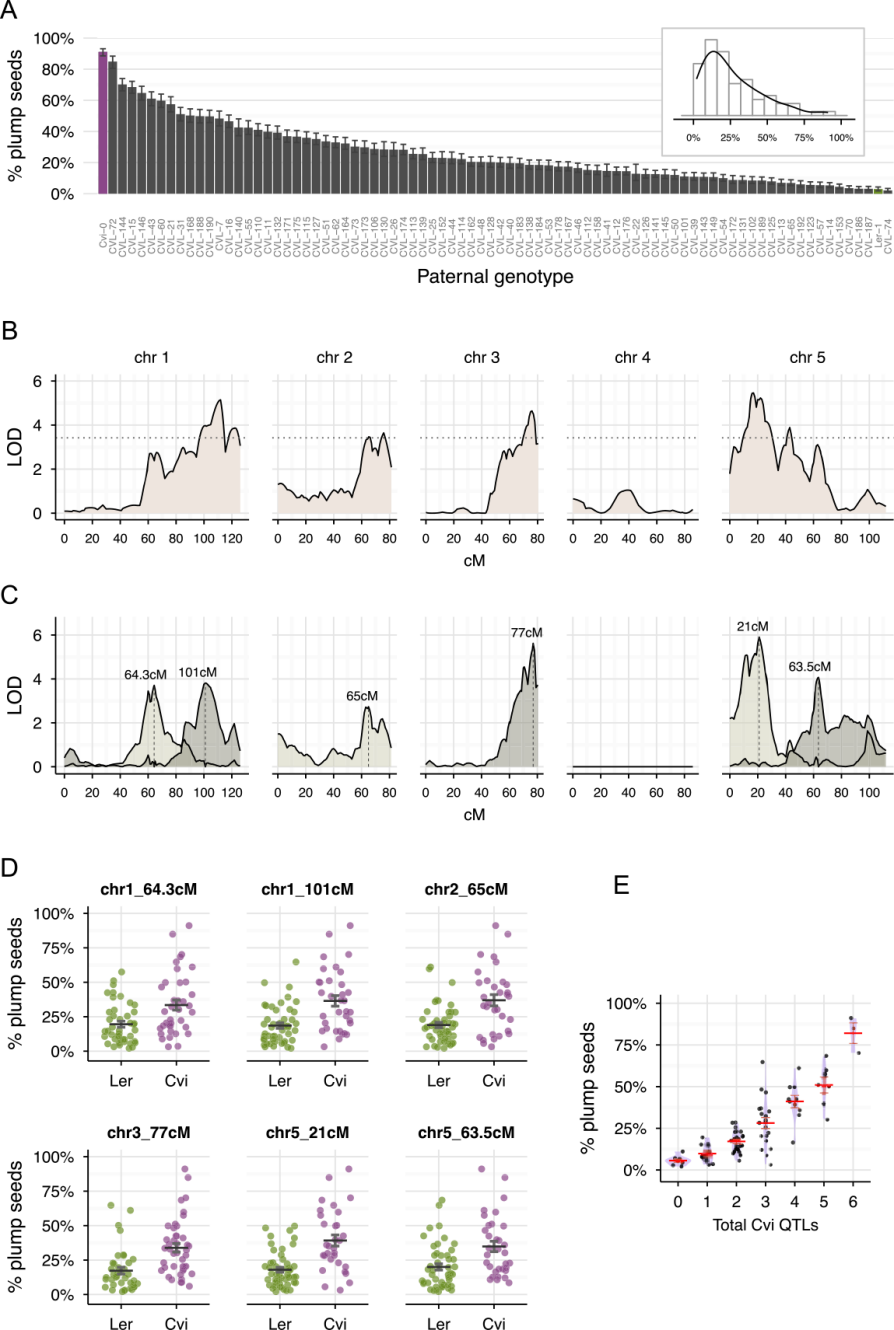
QTL analysis demonstrates that six loci contribute independently to *mea* seed rescue. (A) Percentage of viable plump seeds derived from crosses between homozygous *mea-1*/*mea-1* and pollen from a Ler/Cvi RIL population. Error bars indicate 95% binomial confidence intervals. The inset is a histogram representation of the same data. (B) Standard QTL interval mapping. The dotted line represents the 1% significance LOD threshold. (C) Bayesian estimates for the localization of each of the six QTLs from a multiple QTL model. The values above the peaks represent the best estimate for the localization of the QTL. (D) Effect of individual QTLs. In each subpanel the percentage of plump seeds of all the 80 RILs as in panel A are shown, but sorted according to the genotype (Cvi-0 or Ler) at the respective QTL. The black horizontal line and error bars represent the mean and standard errors. (E) Cumulative effects of Cvi-0 QTLs. The percentage of plump seeds of all the 80 RILs is shown, but sorted according to the total number of Cvi-0 alleles in the six QTLs. The red horizontal lines represent the mean and standard errors. See also Table 1.

We crossed homozygous *mea*/*mea* plants (generated using an inducible MEA-glucocorticoid receptor system) with pollen from 80 Cvi-0/Ler recombinant inbred lines (RILs) for which a detailed genetic map is available [38]. The percentage of plump seeds that originated from these crosses followed a continuous distribution (Fig 3A), indicating that the rescue of *mea* seeds is a polygenic trait. The broad-sense heritability H^2^ (the percentage of total phenotypic variance that can be explained by genetic factors) is 85%, indicating that *mea* seed rescue is under strong genetic control. We used maximum likelihood standard interval mapping to identify regions that are significantly associated with *mea* seed rescue. As expected from the continuous phenotype distribution, we identified multiple QTL peaks on several chromosomes (Fig 3B). Using a multiple-QTL approach [39], we narrowed down these regions to six QTLs, located on chromosome 1 (64.3cM and 101cM), chromosome 2 (65cM), chromosome 3 (77cM) and chromosome 5 (21 and 63.5cM) (Fig 3C). The six QTLs contribute independently to seed rescue (i.e. there was no evidence for epistatic interaction between QTLs) and together explain 73.1% of the phenotypic variance. Each QTL has a relatively small effect and explains a small proportion (5–11%) of the overall phenotypic variation (Fig 3D and Table 1). Nevertheless, the effect of multiple QTLs increases exponentially: every additional Cvi-0 QTL increases the rescued seed rate by roughly 50% (e.g. pollen donors with two, three or four Cvi-0 QTLs generate on average 18%, 28% or 42% plump seeds, respectively) (Fig 3E).

**Table 1:** Location of the Cvi-0 QTLs identified using the RIL population.

Chr	Genetic position (cM)	Approximate physical position (Mbp)	Explained variance
1	64.3	15.6	6.2%
1	101	24.6	6.4%
2	65	15.8	4.5%
3	77	18.7	10%
5	21	5.1	10.6%
5	63.5	15.5	6.9%

**Fig 4:**
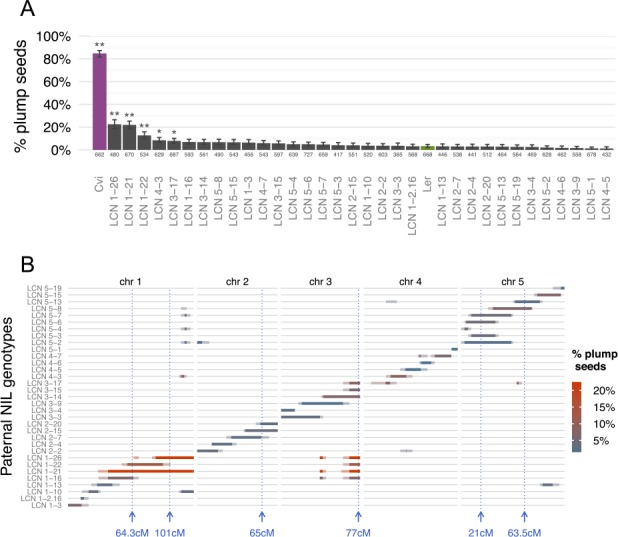
A Cvi/Ler NIL population confirms the polygenic architecture of *mea* rescue. (A) Percentage of viable plump seeds in crosses between homozygous *mea-1*/*mea-1* and pollen from 33 Cvi/Ler NILs. The numbers under the bars represent the number of seeds sampled. Error bars denote 95% binomial confidence intervals. The asterisks indicate that the proportions are significantly different from the crosses with Ler pollen (Bonferroni-corrected one-sided binomial tests, * p < 0.01, ** p < 0.001). (B) Genetic map of the Cvi-0 introgressions in each of the 33 NILs [40]. The solid boxes correspond to the Cvi-0 segments. The thin grey lines indicate the position of Ler segments. The colour scale represents the percentage of viable plump seeds as in panel A. The blue lines indicate the position of the six QTLs identified using the RIL population.

We then crossed *mea/mea* homozygous plants with pollen from a population of Cvi-0/Ler near-isogenic lines (NILs) [40]. Unlike RILs, which have mosaic genomes with a similar proportion of Ler and Cvi-0 genetic backgrounds, these NILs contain only one or a few small introgressions of Cvi-0 in an otherwise homogenous Ler genetic background. We used 33 NILs that together cover 93–98% of the genome with isogenic Cvi-0 fragments. While Cvi-0 pollen gave rise to 85% *mea* plump seeds, almost all the NILs showed no significant differences from Ler (3% viable seeds) (Fig 4A). The three NILs that clearly showed an effect (13–23% viable seeds) actually contain multiple Cvi-0 fragments that overlap two or three of the identified QTLs (Fig 4B). Together, the RIL and NIL analyses suggest the existence of at least six loci in Cvi-0 that contribute to the rescue of *mea* seeds.

We also scored seed abortion in the F2 progeny of a cross between *mea-2* and C24 (genotyped at 14 markers throughout the genome). Despite the low statistical power caused by the segregation of *MEA* in this population, we found evidence for one QTL at the bottom of chromosome 1 that could explain 15% of the observed phenotypic variation (S4 Table). Thus, even this analysis with limited power identified one of the loci on chromosome 1 that was mapped using RILs and NILs.

### Bulk segregant sequencing (Bulk-Seq) analysis confirm the location of the Cvi-0 QTLs

To independently validate the results of the QTL analyses, we developed a novel method for mapping parent-of-origin effects using whole-genome sequencing. The strategy is to create an F2 population that contains one set of chromosomes from one parent but inherits two segregating sets from the other parent. These two sets should have opposing effects in pre- or post-fertilisation fitness or viability, so that they will not be equally transmitted. DNA is then extracted from pools of viable F2 seedlings, and whole-genome sequencing is used to identify genomic regions that exhibit biased transmission of the two segregating paternal (or maternal) genotypes.

**Fig 5:**
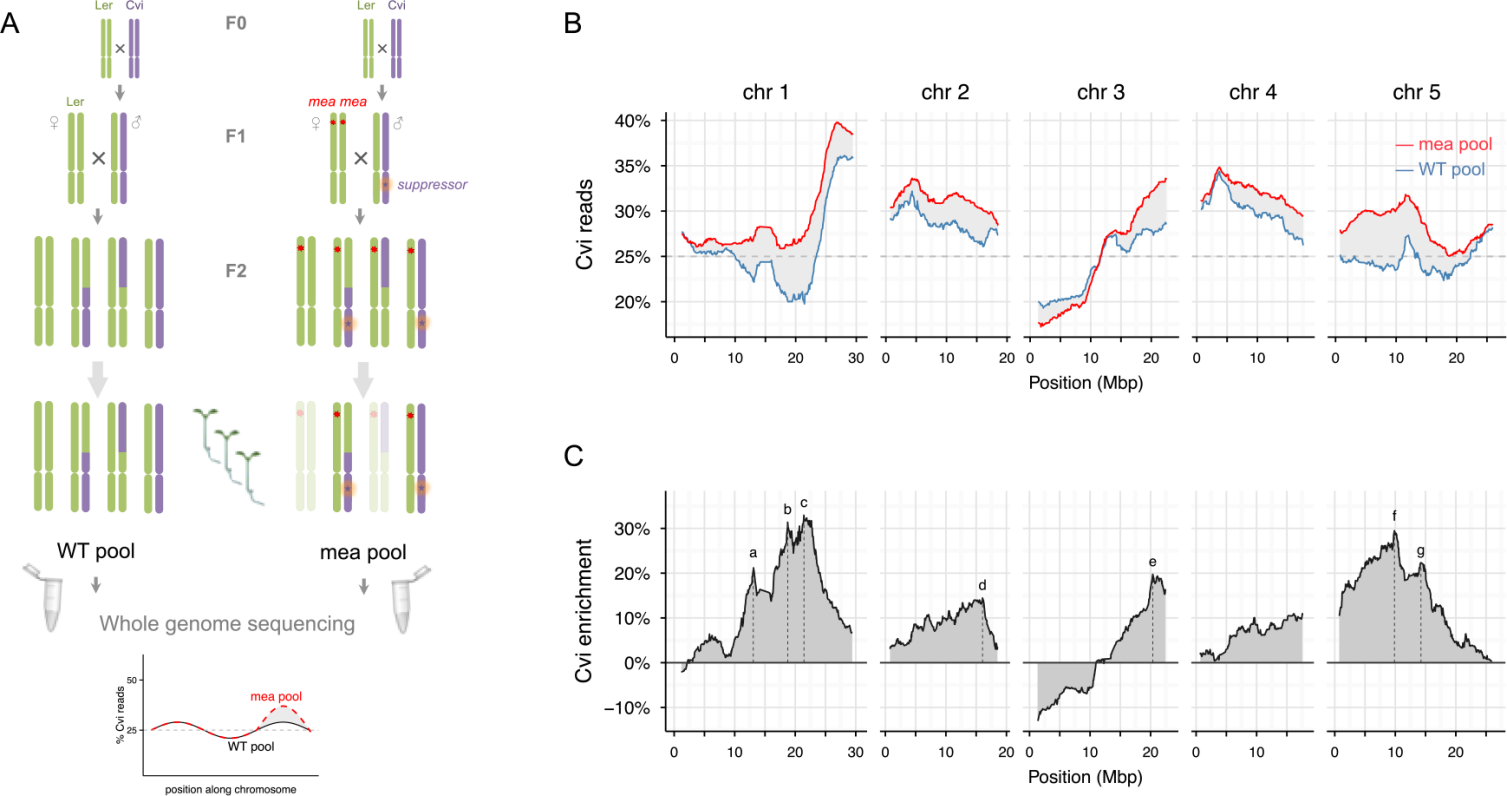
Bulk-Seq analysis to map parent-of-origin effects. (A) Schematic representation of the procedure for Bulk-Seq. (B) Relative proportion of Cvi-0 reads in the *mea* (red) and WT (blue) pools. The dashed line represents the expected 25% average proportion of Cvi-0 reads in the WT pool in the absence of segregation distortion. (C) Enrichment of Cvi-0 reads in the *mea* pool relative to the WT pool. The dotted lines indicate the position of the main peaks; see also Table 2. See S4 Fig for plots of the three independent replicates.

In this case, we took advantage of the differential survival of *mea* seeds depending on the inheritance of Cvi-0 against Ler paternal alleles. First, we generated F1 hybrid plants by reciprocally crossing Ler and Cvi-0 plants (Fig 5A). The Ler/Cvi-0 hybrids were then used to pollinate (1) Ler plants and (2) homozygous *mea/mea* plants (Ler background). The resulting F2 progenies will therefore exclusively inherit Ler chromosomes from the mother (with and without *mea*) but different combinations of Ler and Cvi-0 alleles from the father (due to recombination and segregation of chromosomes during male gametogenesis in the F1 plant). Only *mea* F2 seeds that inherit Cvi-0 alleles that rescue *mea* are able to generate viable seedlings: therefore, genomic regions that are linked to these Cvi-0 alleles will be predominantly transmitted to viable F2 plants. We can identify these by sequencing pools of viable plants and quantifying the relative proportion of Cvi-0 and Ler SNPs throughout the genome. To account for biases in the paternal transmission of Cvi-0 and Ler SNPs that occur independently of *mea*, we determined the transmission of Cvi-0 SNPs in a control cross using wild-type Ler instead of *mea* plants. In this wild-type (WT) control ('WT pool'), we expect the percentage of Cvi-0 reads throughout the genome to be close to 25%; in the *'mea* pool', regions that are associated with *mea* rescue will be enriched in Cvi-0 reads (up to 50%).

**Table 2:** Peaks of Cvi-0 enrichment in the Bulk-Seq analysis.

Peak	Chr	Position (Mbp)	Individual replicates	Cvi enrichment
a	1	13.069	13.517,—, 13.000	22%
b	1	18.754	18.792, 19.322, 20.005	31%
c	1	21.444	22.208, 21.810, 21.170	33%
d	2	16.054	14.735,—, 14.625	14%
e	3	20.370	21.273, 21.963, 20.232	20%
f	5	9.865	8.951, 10.559, 8.656	30%
g	5	14.239	14.807,—, 13.994	22%

We pooled genomic DNA from a total of 2400 viable *mea* seedlings and 1400 WT seedlings in three biological replicates. We then used a dataset of known Ler and Cvi-0 polymorphisms [41,42] to estimate the proportion of Cvi-0 reads throughout the genome (Fig 5B and S4 Fig). In the WT pool, there is clear evidence for segregation distortion in several genomic regions, including a low proportion of Cvi-0 reads at the top of chromosome 3 and the middle of chromosome 1, and a high proportion at the bottom of chromosome 1 and the top of chromosomes 2 and 4. Most of these regions were previously shown to exhibit segregation distortion in crosses between Ler and Cvi-0 [38]. To identify the regions that are associated with *mea* seed rescue, we calculated the difference in the proportion of Cvi-0 reads between the *mea* and WT pools (Fig 5C). There was an overall increase in Cvi-0 reads throughout the genome in the *mea* pool, likely reflecting the highly polygenic nature of *mea* seed rescue; but the enrichment in Cvi-0 reads was particularly pronounced in the middle and bottom of chromosome 1, the bottom of chromosomes 2 and 3, and in the top and middle of chromosome 5: in these regions there was an increase of 15–30% in the proportion of Cvi-0 alleles relative to the WT pool (Fig 5C, Table 2). These peaks were reproducible between the three biological replicates (S4 Fig). Each of the peaks identified by Bulk-Seq is located in the vicinity of the QTLs identified by the RIL-QTL analysis (Table 1); some of the peaks (particularly b, d, and g) are also close to SNPs that were identified by the GWAS analysis as associated (although non-significantly) with *mea* rescue (Fig 2D). Taken together, the Bulk-Seq analysis provides strong support to the existence and predicted location of the multiple Cvi-0 alleles that underlie the rescue of *mea* seeds.

## Discussion

Our results demonstrate that there is a pool of hidden variation in the paternal regulation of seed development in *Arabidopsis*. This paternal variation is released upon maternal loss of *mea*, suggesting that the maternal genome actively buffers the manifestation of paternal effects during seed development. While in the past the effects of the paternal genotype on seed growth were found to be very small or non-existent [23–30], our results clearly indicate that paternal effects exist but are buffered by the maternal genome. This observation is consistent with predictions of the paternal conflict theory, which proposes that the maternal genome counteracts the effect of paternally inherited alleles that would otherwise place extra demands on seed growth.

We hypothesize that, in a maternal Ler background, the (potential) paternal growth demands of Cvi-0 and C24 are lower than the ones of most other accessions (including Ler itself). Upon maternal loss of the buffering mechanism mediated by *MEA*, the paternal growth demands of Ler (and most accessions) lead to excessive seed growth, resulting in *mea* seed collapse; however, the paternal growth demands of Cvi-0 and C24 are not as strong and allow *mea* seeds to complete development.

Interestingly, paternal effects on *mea* seed development are, in turn, dependent on the maternal genetic background: we showed that C24 paternal alleles can strongly rescue *mea* seeds in a maternal Ler or C24 background, but this rescue is much weaker in a Cvi-0 maternal background (Fig 1B). This indicates that there are multiple reciprocal interactions between maternal and paternal alleles in the regulation seed growth.

In many ways, this paternal variation is a classic example of cryptic genetic variation (CGV). Natural genotypes often harbour extensive CGV that is only released upon severe environmental or genetic perturbations [43–45]. Typical examples of CGV include variation in the number of *Drosophila* bristles in a *scute* mutant background [46], inflorescence architectures in maize crossed to its wild ancestor teosinte [47], body size in oceanic stickleback upon exposure to low salinity environments [48], or genetic background-dependent phenotypic variation upon disruption of the heat shock protein Hsp90 in *Drosophila* and *Arabidopsis* [49,50]. CGV usually has no or little effects on phenotypical variation, but it can modify phenotypes under atypical environmental conditions or following the introduction of novel alleles. By acting as a standing pool of genetic information, CGV has been hypothesized to play an important role in adaptation and the evolution of novel characters [51,52]. One explanation for the origin of CGV is that as new mutations appear, their potential phenotypic effect is suppressed by existing buffering mechanisms [44]. During *Arabidopsis* seed development, *MEA* could act as a buffering mechanism that prevents the expression of mutations that would otherwise disrupt the balance of paternal genomes.

Another possibility that could explain the hidden paternal variation is the predominantly self-fertilizing behaviour of *Arabidopsis*. Although imprinting can be maintained in species with a low outcrossing rate [53,54], high kin genetic relatedness is predicted to decrease the intensity of parental conflict [55]. The transition of *Arabidopsis* from an outcrossing to a self-fertilizing species around one million years ago [56], could have resulted in an erosion of the functional importance of imprinting mechanisms and the strength of the parental effects. This could make *Arabidopsis* seeds more resistant to unbalanced crosses and mask the manifestation of parental effects. Supporting this hypothesis, *Arabidopsis* seeds are unusually tolerant of unbalanced interploidy crosses [1,6].

Our genetic analyses demonstrate that multiple loci contribute independently to the paternal rescue of *mea* seeds, but the effect of each individual locus is small. We predict that the underlying genes encode factors that fine-tune embryo and endosperm growth during the early stages of embryogenesis, particularly endosperm cellularization and the transition from radial (globular stage) to bilateral (heart stage) embryo symmetry. We showed that the rescue is directional (e.g. Cvi-0^*mea*^ x Ler seeds abort, but Ler^*mea*^ x Cvi-0 seeds are rescued). This suggests that the rescue is parent-of-origin-specific, and should therefore be meditated by imprinted genes. However, at this point we cannot distinguish whether the underlying alleles are paternally expressed or paternally repressed in Cvi-0. The MADS-box gene *PHE1*, a paternally expressed gene that is a direct target of MEA, is located close to the QTL peak at 101cM in chromosome 1 (peaks b and c in the Bulk-Seq analysis). *phe1* mutants develop slightly lighter seeds and can partially rescue *mea* seeds [57–59], suggesting that variation in *PHE1* may underlie this QTL.

*mea* seeds can be paternally rescued by the *ddm1* mutant or an anti-sense *met1* line [19,60]. Such lines have lower levels of CG methylation, which has been hypothesised to act antagonistically to H3K27 trimethylation to regulate the expression of paternally-derived alleles [61]. Interestingly, Cvi-0 has lower levels of CG methylation in embryos and endosperm than Ler or Col-0 [11]. However, we found no correlation between *mea* seed rescue and global levels of CG methylation in vegetative tissues of over 50 *A*. *thaliana* accessions [62].

The overgrowth phenotype of *mea* seeds strongly resembles the phenotype of interploidy crosses where the ploidy of the male is higher than that of the female [6,63], suggesting that *MEA* is an important contributor to maternal genome dosage. Accordingly, paternal excess crosses can be rescued by increasing the expression of *MEA* [63], while *mea* seeds are viable in maternal excess crosses [64]. Interestingly, hypomethylated pollen can also rescue seeds resulting from unbalanced crosses where the ploidy of the male is higher than the female [65], confirming the existence of overlaps between mechanisms that regulate parental dosage, *Polycomb* activity, and DNA methylation in developing *Arabidopsis* seeds.

Overall, we demonstrate here that there is a large pool of hidden intra-specific variation in the paternal control of seed development. Recent transcriptome studies have shown that 5–15% of *A*. *thaliana* and maize imprinted genes have allele-specific imprinting [11,66,67], while *MEA* has been found to be under positive selection in the genus *Arabidopsis* [68–70]. This suggests that the balancing of parental information during seed development is a very dynamic evolutionary process, and provides strong support to the parental conflict theory for the evolution of imprinting. Importantly, this standing pool of cryptic genetic variation in wild and domesticated species could have important uses in plant breeding programs [71] that aim to regulate seed size or overcome inter-specific hybridizations [72–75].

## Materials and Methods

### Plant material and growth conditions

The *mea-1* and *mea-2* [21], *fis2-1* [76] and *fie* (SALK_042962) [77,78] mutants, as well as the RIL and NIL Ler/Cvi populations [38,40] were previously described. The Landsberg *erecta* (Ler-1), Cape Verde Islands (Cvi-0), and C24 accessions used in this study are derived from lines N22618, N22614, and N22620, respectively, and were a gift of Ortrun Mittelsten Scheid (GMI Vienna). All plants were grown on standard soil (ED73, Einheitserde, Germany) in a greenhouse chamber with 16h light at 20°C and 8h dark at 18°C with an average of 60% humidity.

### Seed and embryo analyses

For seed viability assays, individually crossed siliques were harvested 1–2 days before dehiscence; seeds were then examined under a stereomicroscope and categorised as plump or aborted based on shape, size, and colour. For embryo quantification, seeds at different stages of development were fixed overnight at -20°C with 90% acetone, cleared with chloral hydrate/water/glycerol (8:2:1 w/v/v), and analysed under a Leica DMR microscope.

### Introgression of *mea* into Cvi-0 and C24

For the generation of Cvi-0^*mea/MEA*^ and C24^*mea/MEA*^ lines, *mea*-*2/MEA* plants (Ler background) were used to pollinate Cvi-0 and C24. The F1 progeny was selected in Murashige and Skoog (MS) medium supplemented with 50μg/ml kanamycin (*mea-2* is marked by a kanamycin resistance gene) and backcrossed to Cvi-0 and C24, respectively. Individual backcrossed (BC1) kanamycin-resistant plants were used to pollinate Cvi-0 or C24 and generate independent BC2 lines. Backcrossing was performed for another four generations; BC6 plants were genotyped with a sequence length polymorphism marker linked to the *MEA* locus *(*3.286 Mbp distance) using primers 5'-AATTGAAGCTTTTCTGC-3' and 5'-AGAAAATGAAAAACTTATGG-3' to select plants with homozygous Cvi-0 introgressions close to *mea*. Seed abortion was scored in the progeny of a single BC6 individual from each of three independently generated Cvi-0^*mea/MEA*^ and C24^*mea/MEA*^ lines.

### Segregation analyses

Cvi-0, Hs-0, C24, and Lom-1 were crossed with pollen from *mea-2*, the F1 populations were allowed to self-fertilize, and DNA was extracted from viable seedlings. *mea-2* plants were genotyped using primers 5'-CCAATGCACAAATCGACAATG-3' and 5'-CACCAAGAGTGCCATCTCCA-3' (WT genomic DNA), and 5'-CGATTACCGTATTTATCCCGTTCG-3' (*Ds* insertion tightly linked to the *mea-2* allele [21]).

### Generation of homozygous *mea*/*mea* seeds

To obtain *pMEA*::*MEA-GR*, an 8.6 kb genomic fragment (4.4kb upstream region + 4.2 kb *MEA* ORF) was amplified from the previously generated plasmid pCambia 1381Z [20] using primers proMEA-BPfor (AAAAAGCAGGCTCACTAAGATATGTTGGGTC) and MEA-BP-GRrev (AGAAAGCTGGGTCTGCTCGACCTGCCCGA), and recombined into pDONR207 using Gateway cloning (Invitrogen). The resulting entry vector was subsequently recombined into *pDEST-GR* (*pARC146* without 35*S* promoter) [79], and the fusion between *MEA* and the *GR* domain sequence was confirmed by sequencing. The construct was introduced into the *Agrobacterium* strain GV3101::pMP90, and transformed into Ler plants using floral dip [80]. Lines expressing the construct were crossed with *mea-1*, and the offspring was continuously watered with 10 μM dexamethasone (DEX) (Sigma cat. D1756) to identify reduced seed abortion and thus rescue by the construct. Several lines were identified that complemented the *mea-1* seed abortion phenotype to a large extent only in the presence of 10 μM DEX. The best complementing line (line 18) was used to raise the homozygous *mea-1*/*mea-1* plants used in this study.

### RIL QTL analyses

A total of 47,619 seeds derived from crosses between homozygous *mea-1/mea-1* plants and 80 Ler/Cvi RILs were scored. The genotype map for these lines included 144 markers [38,81] and was kindly provided by Joost Keurentjes (Wageningen University and Research Centre). Broad-sense heritability was estimated with an analysis of variance of a linear mixed-effects model, using the lmer function of the 'lme4' R package [82]. Means and confidence intervals for each RIL were estimated using a binomial regression, and normalised using a cubic root transformation. QTL analyses were performed using the 'R/qtl' R package [39]. Genotype data across the genome was estimated using multiple imputation at a 1cM density, and interval mapping was calculated using standard maximum likelihood estimation; the LOD (logarithm of odds) threshold at 1% was calculated using a permutation test with 5000 replicates. Multiple-QTL models were selected using a combination of automated stepwise model selection and iterative individual QTL location refinement as implemented in 'R/qtl'; the penalized LOD scores used to guide model selection were derived using a permutation test with 1000 replicates of a two-dimensional, two-QTL genome scan. The best fit-model we identified had six loci and no epistatic interactions between the QTLs. Finer localizations of each of the QTLs of the best-fit model along the chromosomes were estimated using a Bayesian approach, as implemented in the bayesint function of 'R/qtl'. The effect of individual QTLs was estimated as the proportion of phenotypic variance they explain. The approximate physical location was estimated using the physical location of genetic markers [38,81].

For the *mea-2* x C24 QTL analysis, an F2 population of 247 individuals was generated and genotyped using 14 sequence length polymorphism markers. An ANOVA regression was calculated for each marker: the values on S4 Table are the p-values for a regression made using a subset of 35 homozygous *mea/mea* plants.

### Preparation of DNA pools for Bulk-Seq

Ler and Cvi-0 were reciprocally crossed to generate Ler/Cvi-0 hybrids. These were then used to pollinate homozygous *mea*/*mea* or Ler plants. Three independent replicates were generated, each using different parental individuals and at different days. In total, around 10,000 and 4,000 F2 seeds were generated from *mea*/*mea* and Ler mothers, respectively. Seeds were surface sterilised for 10 minutes using 1% sodium hypochlorite, washed extensively with water, and sown in MS medium. After 10–14 days of growth at 22°C, leaves from viable seedlings were collected (825, 800, and 775 seedlings for each of the WT pool replicates; 600, 400, and 400 individuals for each of the *mea* pool replicates). DNA was extracted in groups of 50 leaves using the DNeasy Plant Mini Kit (Qiagen cat. 69104), and quantified using the Qubit dsDNA HS Assay kit (Life Technologies cat. Q32854). DNA from the different extractions was pooled in equi-amounts, precipitated using sodium acetate and isopropanol, and resuspended in 25 μl TE buffer for each replicate at a final concentration of 100–150 ng/μl.

### Library preparation and sequencing

The TruSeq DNA Sample Prep Kit v2 (Illumina) was used in the succeeding steps. DNA samples (1 μg) were sonicated and the fragmented DNA samples end-repaired and polyadenylated. TruSeq adapters containing the index for multiplexing were ligated to the fragmented DNA samples. The ligated samples were run on a 2% agarose gel and the desired fragment length was excised (50bp +/- the target fragment length). DNA from the gel was purified with MinElute Gel Extraction Kit (Qiagen). Fragments containing TruSeq adapters on both ends were selectively enriched with PCR. The quality and quantity of the enriched libraries were validated using Qubit (1.0) Fluorometer and the Caliper GX LabChip GX (Caliper Life Sciences). The product is a smear with an average fragment size of approximately 260 bp. The libraries were normalized to 10nM in Tris-Cl 10 mM, pH8.5 with 0.1% Tween 20. The TruSeq SR Cluster Kit (Illumina) was used for cluster generation using 5 pM of pooled normalized libraries on the cBOT. Sequencing of single reads was performed on one lane of the Illumina HiSeq 2000 using the TruSeq SBS Kit v3-HS (Illumina Inc, USA).

### Read mapping and SNP segregation analyses

Reads were quality-checked using FastQC (http://www.bioinformatics.babraham.ac.uk/projects/fastqc) and trimmed with the FASTX-Toolkit (http://hannonlab.cshl.edu/fastx_toolkit/). Processed reads were aligned to the TAIR10 version of the *Arabidopsis* genome (Col-0 accession) using Bowtie2 [83] and the SAMtools package [84]. SNPs were called using the mpileup command from SAMtools. Known SNPs from Ler-1 and Cvi-0 [41,42] were retrieved from http://1001genomes.org/data/MPI/MPISchneeberger2011/releases/current//Ler-1/Marker/Ler-1.SNPs.TAIR9.txt (461,070 Ler-1/Col-0 SNPs) and from http://signal.salk.edu/atg1001/data/Salk/quality_variant_filtered_Cvi_0.txt (657027 Cvi-0/Col-0 SNPs). After removing common Ler/Cvi-0 SNPs, we obtained a list with 765,643 SNPs. This list was combined with the SNP calls from the bulk sequencing dataset; for each of the datasets (three replicates of each pool), we then removed positions that were (1) ambiguous in the reference sequence; (2) did not match between the Bulk-Seq samples and the published SNPs; and (3) had very high coverage (above the 99% percentile of a Poisson distribution with λ = median coverage of the sample). These quality-filtering steps resulted in an average of 390,000 SNPs for each of the six datasets. The three replicates were combined by summing up the number of reads at each SNP position. SNP positions that had no Ler or no Cvi-0 reads in the combined dataset were discarded, as were the top and low 1% quantiles of coverage. This resulted in a final combined dataset of 352,491 SNPs with an average read coverage of 16 Ler and 6 Cvi-0 reads, respectively (S3 Table). The same quality filtering steps were performed in each of the three replicates. To calculate the relative proportion of Cvi-0 and Ler reads across the genome, we first summed the read counts of groups of 50 neighbouring SNPs across the genome using a rolling window. We then calculated the proportion of Cvi-0 and Ler reads, and the relative enrichment of Cvi-0 reads in the *mea* pool relative to the WT pool:

$$\%\;\text{Cvi enrichment}=\frac{\%\;{\text{Cvi}}^{\;mea\;\text{pool}}-\%\;{\text{Cvi}}^{\;\text{WT pool}}}{\%\;{\text{Cvi}}^{\;\text{WT pool}}}$$

Finally, we smoothed Cvi enrichment across neighbouring positions by computing the median of Cvi-0 enrichment with a rolling window of 100 SNPs.

### Genome-wide association mapping

We used *mea-2* to pollinate 167 *Arabidopsis* accessions obtained from the Nottingham Arabidopsis Stock Centre (NASC) or as a kind gift from Ortrun Mittelsten Scheid (Gregor Mendel Institute) and Takashi Tsuchimatsu (University of Zurich). Accessions are detailed in S1 Table. The F1 progeny was selected in MS medium supplemented with 50μg/ml kanamycin and allowed to self-fertilize. We collected individual fruits 1–2 days before dehiscence and visually scored seed phenotypes; some fruits had a high proportion of autonomous seeds (*mea* autonomous endosperm development depends on the genetic background [85]); to avoid a bias in the aborted/plump seed ratio calculations, we did not use fruits that had more than 8% autonomous seeds (225 out of 2046 fruits; there was no correlation between *mea* rescue and autonomous seed development). We discarded three outliers (accessions with a low number of scored seeds or relatively high percentage of autonomous seed development) and obtained a final dataset consisting of 93,884 seeds from 164 accessions. Genotypic information (250k snp data v3.06) was downloaded from https://cynin.gmi.oeaw.ac.at/home/. For correlation analysis with the dataset of 107 phenotypes [35], we calculated Pearson correlations and corrected p-values for multiple testing using the Benjamini-Hochberg false discovery rate procedure.

Genome-wide association mapping was performed with compressed mixed linear models [86] implemented in the 'GAPIT' R package [87] and in the web-based GWAPP portal [88] using the proportion of plump seeds as a phenotype (cubic root normalised). Plots were generated using the 'ggplot2' R package [89].

## Supporting Information

S1 Fig Embryo development in WT and *mea* seeds. The percentage of embryo stages in Ler and *mea*/*mea* siliques developing 4, 5, 6, 7, and 8 days after pollination with Ler or Cvi-0 pollen. Whereas *mea* ovules pollinated with Ler arrest at the globular stage, the ones pollinated with Cvi-0 can progress to the torpedo stage: however, development is delayed in *mea* embryos (at 7 days after fertilization 70–80% of WT embryos are at torpedo stage, against only 30–35% of *mea* x Cvi-0 embryos). n = 120–290 seeds; gl, globular stage; tr, triangle stage; hr, heart stage; tp, torpedo stage; ws, walking stick stage.
(PDF)

S2 Fig *mea* rescue among seeds derived from self-fertilized F2 homozygous *mea*/*mea* individuals from the Cvi-0 population. The 12 individuals are homozygotes from the F2 Cvi-0 x *mea-2* population described in S1 Table. The numbers under the bars represent the number of seeds sampled. Error bars denote 95% binomial confidence intervals.
(PDF)

S3 Fig Correlation between *mea* rescue and nutrient and flowering time status of different *Arabidopsis* accessions. The horizontal axis shows the percentage of plump seeds obtained in the F2 of crosses between *mea-2* and different *Arabidopsis* accessions (as in S2 Table). The vertical axis shows the *in planta* calcium and magnesium concentrations, and time to flowering under field conditions and under short days and vernalization. The grey lines denote a linear regression; p-values from the Pearson correlation test were corrected for multiple testing using the Benjamini-Hochberg method.
(PDF)

S4 Fig Bulk-Seq analysis of individual replicates. (A) Relative proportion of Cvi-0 reads in the *mea* (red) and WT (blue) pools in each of three biological replicates. (B) Relative enrichment in Cvi-0 reads. See also Fig 5 and Table 2.
(PDF)

S1 Table Segregation of *mea* in F2 populations. (DOCX)

S2 Table Accessions used for the generation of F2 populations with *mea*. % plump seeds correspond to the values depicted in Fig 2A.
(XLSX)

S3 Table Final read coverage and SNPs used for the Bulk_Seq analysis (after quality filtering steps). (DOCX)

S4 Table QTL analysis of an F2 population of C24 x *mea-2*. (DOCX)
