# Supplementary figures and images for: Quantitative Genetics Identifies Cryptic Genetic Variation Involved in the Paternal Regulation of Seed Development
Source: PLoS Genet. 2016 Jan 26;12(1):e1005806. doi: 10.1371/journal.pgen.1005806 (PMC4727937; doi:10.1371/journal.pgen.1005806)

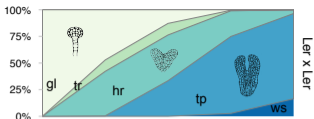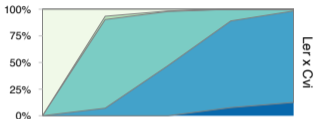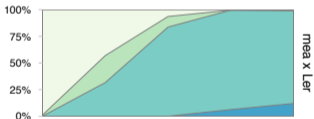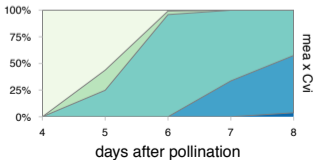

Supplement: S1 Fig — The percentage of embryo stages in Ler and mea/mea siliques developing 4, 5, 6, 7, and 8 days after pollination with Ler or Cvi-0 pollen. Whereas mea ovules pollinated with Ler arrest at the globular stage, the ones pollinated with Cvi-0 can progress to the torpedo stage: however, development is delayed in mea embryos (at 7 days after fertilization 70–80% of WT embryos are at torpedo stage, against only 30–35% of mea x Cvi-0 embryos). n = 120–290 seeds; gl, globular stage; tr, triangle stage; hr, heart stage; tp, torpedo stage; ws, walking stick stage. (PDF) [file pgen.1005806.s001.pdf]

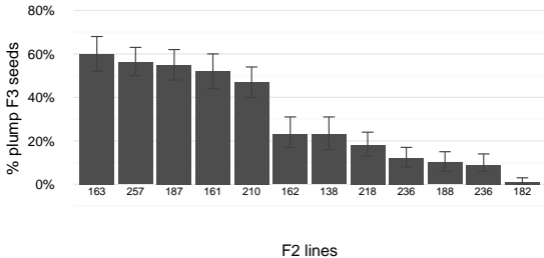

Supplement: S2 Fig — The 12 individuals are homozygotes from the F2 Cvi-0 x mea-2 population described in S1 Table. The numbers under the bars represent the number of seeds sampled. Error bars denote 95% binomial confidence intervals. (PDF) [file pgen.1005806.s002.pdf]

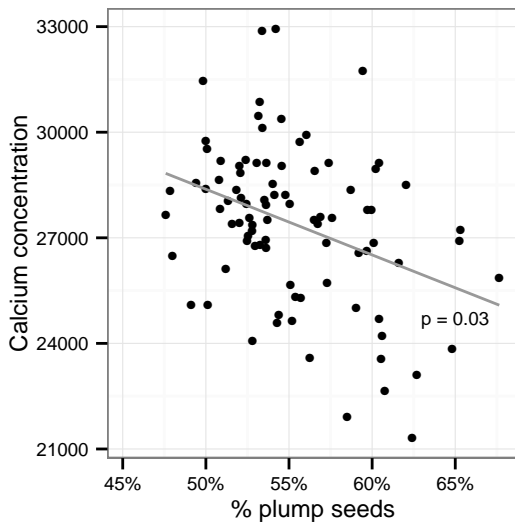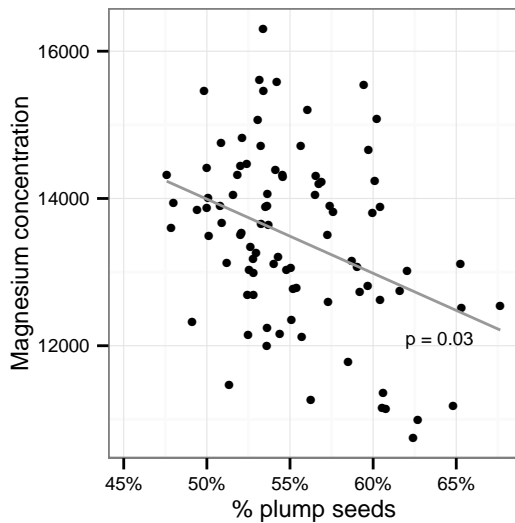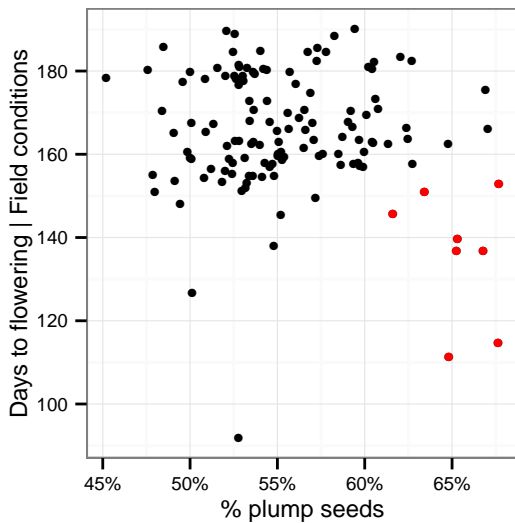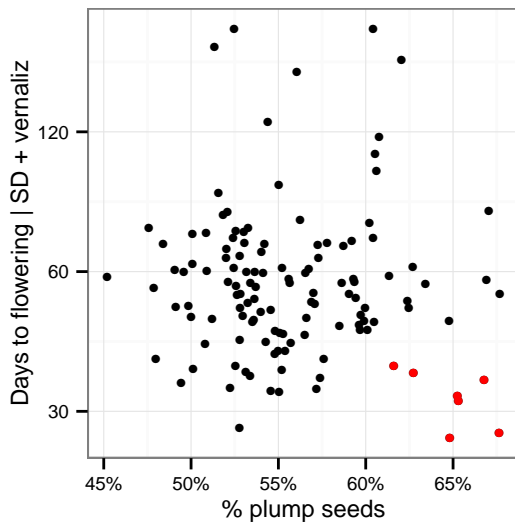

Supplement: S3 Fig — The horizontal axis shows the percentage of plump seeds obtained in the F2 of crosses between mea-2 and different Arabidopsis accessions (as in S2 Table). The vertical axis shows the in planta calcium and magnesium concentrations, and time to flowering under field conditions and under short days and vernalization. The grey lines denote a linear regression; p-values from the Pearson correlation test were corrected for multiple testing using the Benjamini-Hochberg method. (PDF) [file pgen.1005806.s003.pdf]

**A**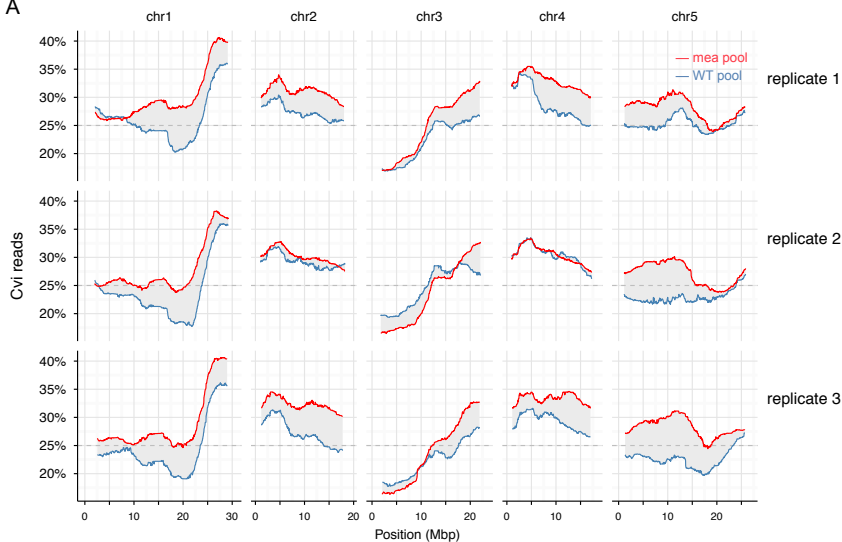**B**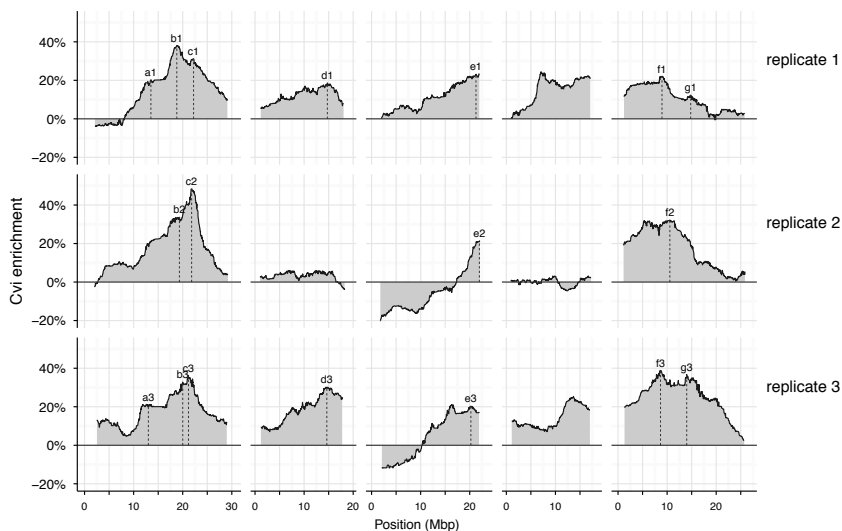

Supplement: S4 Fig — (A) Relative proportion of Cvi-0 reads in the mea (red) and WT (blue) pools in each of three biological replicates. (B) Relative enrichment in Cvi-0 reads. See also Fig 5 and Table 2. (PDF) [file pgen.1005806.s004.pdf]
