# Supplementary material for: Quantitative Genetics Identifies Cryptic Genetic Variation Involved in the Paternal Regulation of Seed Development
Source: PLoS Genet. 2016 Jan 26;12(1):e1005806. doi: 10.1371/journal.pgen.1005806 (PMC4727937; doi:10.1371/journal.pgen.1005806)
Supplement: S1 Table — (DOCX) [file pgen.1005806.s005.docx]

| **F2 population** | ***mea* segregation**  **(WT: heterozygous : homozygous)** | **n** | **% *mea/mea* homozygotes** |
| --- | --- | --- | --- |
| Cvi-0 | 271:224:13 | 508 | 3% |
| C24 | 68:86:16 | 170 | 9% |
| Hs-0 | 58:79:34 | 171 | 20% |
| Lom1-1 | 70:70:15 | 155 | 10% |
