# Supplementary material for: Quantitative Genetics Identifies Cryptic Genetic Variation Involved in the Paternal Regulation of Seed Development
Source: PLoS Genet. 2016 Jan 26;12(1):e1005806. doi: 10.1371/journal.pgen.1005806 (PMC4727937; doi:10.1371/journal.pgen.1005806)
Supplement: S3 Table — (DOCX) [file pgen.1005806.s007.docx]

|  | **Total SNPs** | **Median read coverage** | | | |
| --- | --- | --- | --- | --- | --- |
|  |  | Ler pool | | mea pool | |
|  |  | *Ler reads* | *Cvi reads* | *Ler reads* | *Cvi reads* |
| Replicate 1 | 220652 | 6 | 2 | 6 | 3 |
| Replicate 2 | 240967 | 6 | 2 | 10 | 4 |
| Replicate 3 | 203788 | 6 | 2 | 5 | 2 |
| Combined | 352491 | 14 | 5 | 18 | 7 |
