# Supplementary material for: Quantitative Genetics Identifies Cryptic Genetic Variation Involved in the Paternal Regulation of Seed Development
Source: PLoS Genet. 2016 Jan 26;12(1):e1005806. doi: 10.1371/journal.pgen.1005806 (PMC4727937; doi:10.1371/journal.pgen.1005806)
Supplement: S4 Table — (DOCX) [file pgen.1005806.s008.docx]

| **Marker** | **chr** | **position (cM)** | **p-value** |
| --- | --- | --- | --- |
| uzu5 | 1 | 50 | 0.1535 |
| T23K8-1 | 1 | 93 | **0.0014** |
| uzu7 | 1 | 94 | **0.0004** |
| T8F5-1 | 1 | 95 | **0.0012** |
| ciw2 | 2 | 7 | 0.0590 |
| nga1126 | 2 | 51 | 0.9675 |
| nga361 | 2 | 63.02 | 0.9450 |
| nga162 | 3 | 20.56 | 0.6784 |
| nga6 | 3 | 86.41 | 0.6981 |
| JV30/31 | 4 | 1 | 0.0446 |
| F17N18-1 | 4 | 42 | 0.2517 |
| MXM12-1ME | 5 | 12 | 0.0382 |
| ciw9 | 5 | 70 | 0.9618 |
